# Laser Doppler Vibrometry measurements of human cadaveric tympanic membrane vibration

**DOI:** 10.1186/1916-0216-42-17

**Published:** 2013-02-25

**Authors:** Jason A Beyea, Seyed Alireza Rohani, Hanif M Ladak, Sumit K Agrawal

**Affiliations:** 1 Department of Otolaryngology – Head and Neck Surgery, Schulich School of Medicine and Dentistry, Western University, London, ON, Canada; 2 Biomedical Engineering Graduate Program, Western University, London, ON, Canada; 3 Department of Medical Biophysics, Western University, London, ON, Canada; 4 Department of Electrical and Computer Engineering, Western University, London, ON, Canada; 5 Schulich School of Medicine and Dentistry, Department of Otolaryngology - Head and Neck Surgery, Western University, 339 Windermere Road, P.O. Box 5339, London, ON, Canada

**Keywords:** Laser Doppler Vibrometry, Malleus head fixation, Incudo-stapedial joint separation, Otosclerosis, Tympanic membrane vibration, Conductive hearing loss

## Abstract

**Objective:**

To determine the feasibility of measuring tympanic membrane (TM) vibrations at multiple locations on the TM to differentiate normal eardrums from those with associated ossicular pathologies.

**Design:**

Cadaveric human temporal bone study.

**Setting:**

Basic science laboratory.

**Methods:**

A mastoidectomy and facial recess approach was performed on four cadaveric temporal bones to obtain access to the ossicles without disrupting the TM. Ossicles were palpated to ensure normal mobility and an intact ossicular chain. Laser Doppler Vibrometry (LDV) measurements were then taken on all four TMs. LDV measurements were repeated on each TM following stapes footplate fixation, incudo-stapedial joint dislocation, and malleus head fixation.

**Main outcome measures:**

LDV measurements of TM vibration at the umbo, the lateral process of the malleus, and in each of the four quadrants of the TM.

**Results:**

The best signal-to-noise ratios were found between 2 and 4 kHz, at the umbo, the anterior superior quadrant, the anterior inferior quadrant, and the posterior inferior quadrant. Since our goal was to assess the ossicular chain, we selected the TM locations closest to the ossicular chain (the umbo and lateral process of the malleus) for further analysis. Differences could be seen between normals and the simulated ossicular pathologies, but values were not statistically significant.

**Conclusions:**

LDV measurements are technically challenging and require optimization to obtain consistent measurements. This study demonstrates the potential of LDV to differentiate ossicular pathologies behind an intact tympanic membrane. Future studies will further characterize the clinical role of this diagnostic modality.

## Introduction

Laser Doppler Vibrometry (LDV) is a non-contacting optical technique which can be used to measure tympanic membrane (TM) vibration and middle ear function. This has previously been reported in fresh [1] and embalmed [2] cadaveric human temporal bones, and in live human subjects [3-5]. Although previous studies have analyzed ossicular abnormalities with LDV [3-6], this method is not clinically diagnostic at present in patients with an intact TM and a conductive hearing loss (CHL).

Diagnosis of the specific cause of a CHL in a patient with an intact TM is not presently possible with current testing modalities. However, current audiologic and impedance testing can suggest possible causes. For instance, a type As tympanogram can suggest ossicular fixation, whereas a type Ad tympanogram can suggest ossicular discontinuity. Furthermore, the absence of acoustic reflexes can imply an abnormality of the ossicles. A patient with a CHL and intact acoustic reflexes should be screened for vestibular symptoms and undergo a high-resolution CT scan of the temporal bones to rule out Superior Semicircular Canal Dehiscence Syndrome. At present, an exploratory tympanotomy is required to definitively elucidate the cause of the CHL in these patients with an intact TM and an unremarkable high-resolution CT scan of the temporal bones.

The primary objective of this study was to test the feasibility of non-operative diagnosis of ossicular abnormalities using LDV measurements of tympanic membrane vibration in cadaveric human temporal bones under normal conditions and then following simulated ossicular pathologies. The secondary objective was to assess whether measuring vibrations at a site closer to the ossicular pathology (e.g. lateral process of the malleus) is more diagnostic than the classic position at the umbo.

## Methods

### Cadaveric temporal bone preparations

Six adult cadaveric temporal bones were obtained fresh-frozen within 24 hours after death. Cadaveric materials were donated to the Western University Schulich School of Medicine and Dentistry Department of Anatomy and Cell Biology for the purposes of medical education and research. Permission was granted for the use of the cadaveric temporal bones in the present study. A thawing protocol similar to that of Pennings et al [7] was used in this study. Following thawing, a standard mastoidectomy and facial recess approach were performed on all temporal bones using an operating microscope. Temporal bones and tympanic membranes were kept moist with saline irrigation and suctioning. To access the stapes footplate, the facial recess was widened posteriorly by removal of the mastoid segment of the facial nerve [8]. The pinna and part of the lateral cartilaginous external auditory canal (EAC) was removed from all temporal bones in order to increase exposure. Debris was microdebrided from the EAC, and each tympanic membrane was evaluated to ensure absence of any abnormalities. In two temporal bones, tympanic membrane perforations were present. These two bones were excluded, which resulted in four bones that were included in this study. In two cadaveric temporal bones, the anterior canal wall was prominent, which impaired direct visualization of the anterior TM by the laser Doppler vibrometer. In these bones, an anterior canal wall canaloplasty was performed with a 2 mm diamond burr to improve visualization. The ossicles were palpated through the widened facial recess. Each temporal bone had an intact ossicular chain with absence of malleus head fixation or fixation of the stapes footplate.

### Simulation of ossicular pathologies

The control group consisted of all four normal TMs with intact ossicular chains. There were three simulated ossicular pathology groups: stapes footplate fixation, incudo-stapedial (IS) joint separation, and malleus head fixation. Simulation of ossicular pathologies was performed with the use of an operating microscope.

Stapes footplate fixation was achieved by the placement of ethyl cyanoacrylate glue on the edges of the oval window. Complete fixation was determined by stapes palpation.

The IS joint was separated with a 45 degree pick. To further ensure the absence of contact between the incus and stapes, a malleus head nipper was used to remove the distal portion of the long process of the incus.

The malleus head was fixed by application of ethyl cyanoacrylate glue to the head of the malleus through the mastoid antrum. Complete fixation was determined by malleus palpation.

### Laser Doppler vibrometry measurements

**Figure 1:**
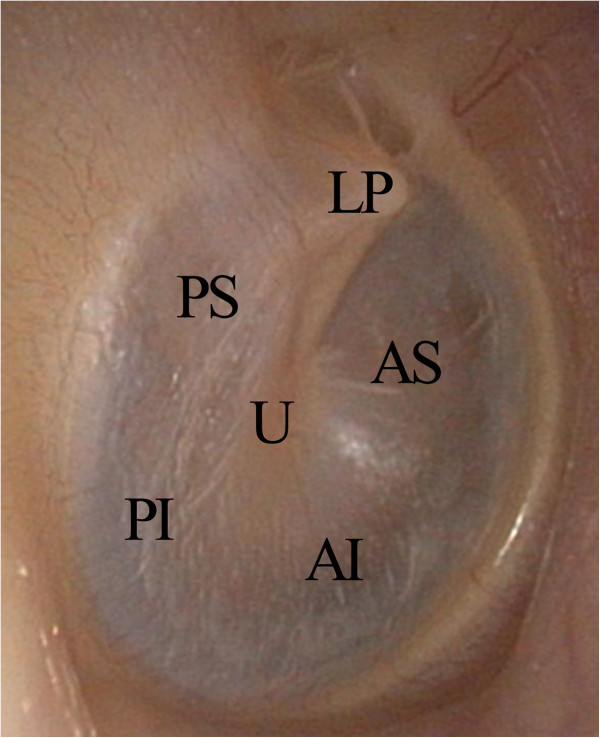
**Locations of laser Doppler Vibrometry measurements on the tympanic membrane.** U = Umbo, LP = Lateral Process of the Malleus, AS = Anterior Superior Quadrant, AI = Anterior Inferior Quadrant, PS = Posterior Superior Quadrant, PI = Posterior Inferior Quadrant.

A Polytec PSV-400 Scanning Laser Doppler Vibrometer with Vibrometer Controller OFV-5000 (Polytec GmbH, Waldbronn, Germany) mounted on a Vibraplane Airmount Isolation table (Kinetic Systems, Inc., Boston, MA, U.S.A.) was used for all LDV measurements. LDV measurements were obtained in all four cadaveric temporal bones from the normal TM with intact ossicular chain, the TM after stapes fixation, the TM after IS joint separation, and the TM after malleus head fixation. For each cadaveric ear in each of the experimental conditions, LDV measurements were taken from six locations on the TM (Figure 1): the umbo, the lateral process of the malleus, the anterior superior quadrant, the anterior inferior quadrant, the posterior superior quadrant, and the posterior inferior quadrant. For each TM location, LDV measurements were taken from nine points arranged in a 3×3 grid, then averaged.

**Figure 2:**
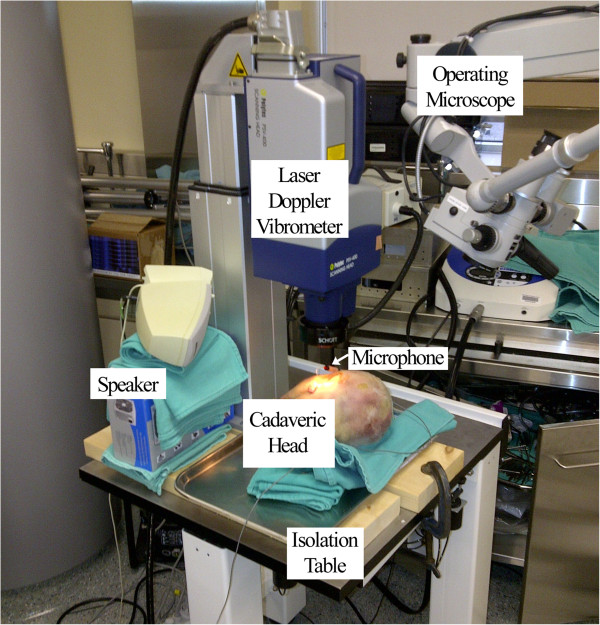
**Experimental laser Doppler Vibrometry setup.** The laser Doppler vibrometer is mounted on an isolation table, adjacent to the speaker. The microphone is placed in the external auditory canal. An operating microscope is available for ossicular inspection, palpation, and modifications.

The TMs were stimulated with a 75-80 dB stimulus from 10 to 8000 Hz with a frequency sweep measured at each increment of 2.5 Hz, delivered through a Harman-Kardon HK-195 speaker (Harman Kardon, Stamford, CT, U.S.A.) placed adjacent to the cadaveric head. Sound pressure was measured with an ER-7C microphone (Etymotic Research, Elk Grove Village, IL, U.S.A.) placed within 2 mm of the TM, and a sound pressure of >75 dB was measured from 200-8000 Hz. LDV measurements were collected within the frequency range of 10–8000 Hz. Measurements were repeated until two comparable tracings were obtained from all TM locations in all experimental groups. Once obtained, the two comparable tracings were averaged to produce one tracing for each TM location in each cadaveric head in each experimental group. Our experimental setup is shown in Figure 2.

### Data analysis

All data were obtained with supplied Polytec software version 8.5 (Polytec GmbH, Waldbronn, Germany). Data were exported to Matlab (Mathworks, Natick, Massachusetts, U.S.A.) for further analysis. To meet our signal-to-noise ratio (SNR) criteria, two conditions must have been met: (1) TM displacement was 10 dB greater than the background TM displacement with stimulus off, *or* TM velocity was 10 dB greater than the background TM velocity with stimulus off, *and* (2) sound pressure was 20 dB SPL (sound pressure level) greater than the background sound pressure with stimulus off. If the data did not meet the SNR, they were excluded from further analysis. Data were plotted normalized to the EAC sound pressure. Displacement versus sound pressure and velocity versus sound pressure across a frequency range of 0.1 to 8 kHz were plotted to reflect prior publications in the literature [3,9]. Raw data is presented (the data were not smoothed). For the umbo and lateral process of the malleus data, a *t*-test was performed at α = 0.05 at all frequencies comparing each of the experimental groups (stapes footplate fixation, IS joint separation, and malleus head fixation) to the normal group using a Bonferonni correction.

## Results

**Figure 3:**
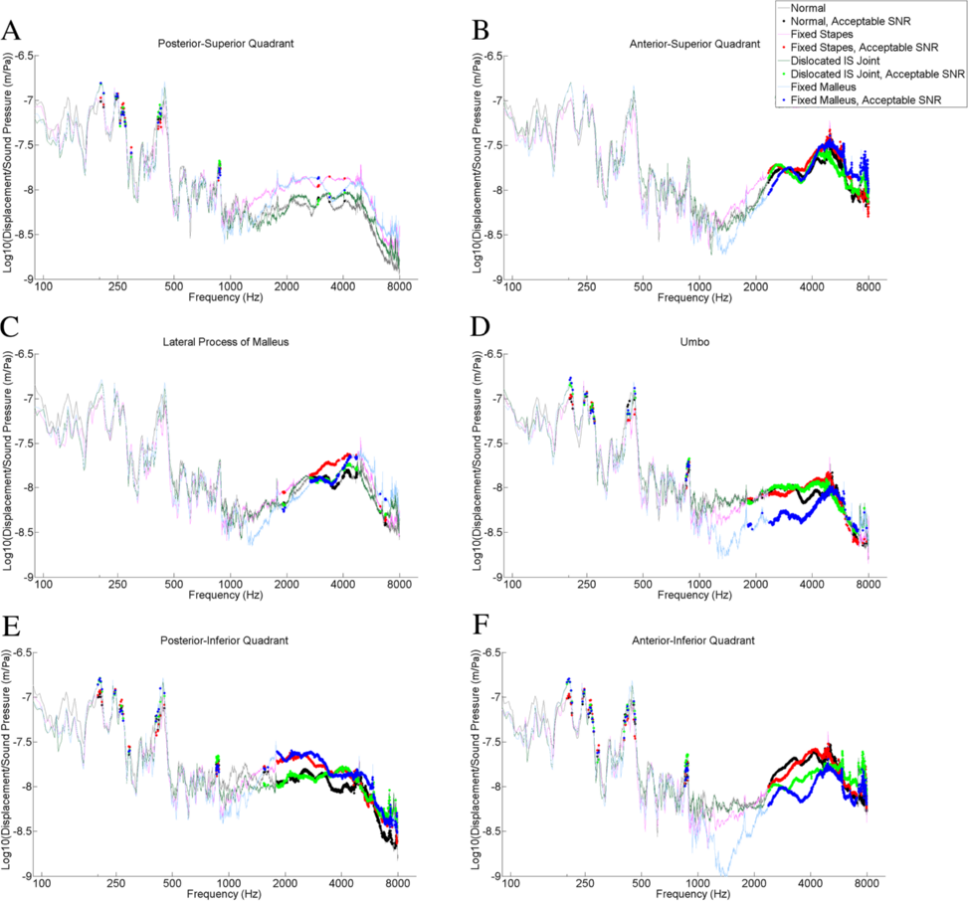
**Logarithm of tympanic membrane displacement normalized to background sound pressure measured from the external auditory canal plotted against the frequency of sound stimulus.** Results are averaged over four bones per experimental group. Tympanic membrane locations were: **A**. Posterior superior quadrant, **B**. Anterior superior quadrant, **C**. Lateral process of malleus, **D**. Umbo, **E**. Posterior inferior quadrant, **F**. Anterior inferior quadrant. Legend: For each tracing, the thin lines represent frequencies at which the SNR (signal to noise ratio) did not meet our criteria (see text). Thick lines represent frequencies at which the SNR met our criteria.

**Figure 4:**
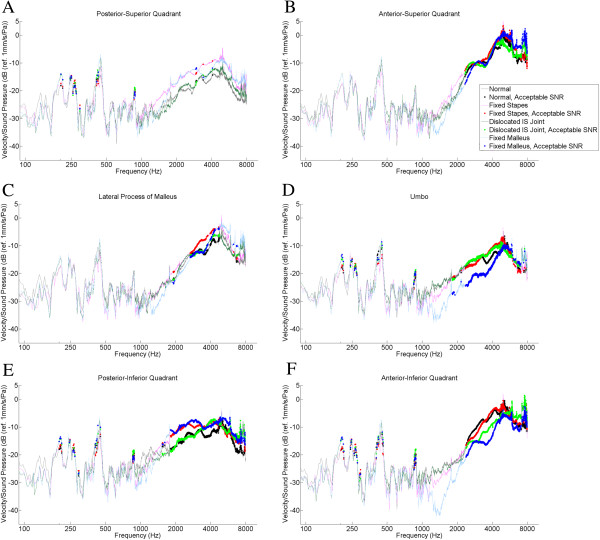
**Logarithm of tympanic membrane velocity normalized to background sound pressure measured from the external auditory canal plotted against the frequency of sound stimulus.** Results are averaged over four bones per experimental group. Tympanic membrane locations were: **A**. Posterior superior quadrant, **B**. Anterior superior quadrant, **C**. Lateral process of malleus, **D**. Umbo, **E**. Posterior inferior quadrant, **F**. Anterior inferior quadrant. Legend: For each tracing, the thin lines represent frequencies at which the SNR (signal to noise ratio) did not meet our criteria (see text). Thick lines represent frequencies at which the SNR met our criteria.

Overall, the best signal-to-noise ratios were found between 2 and 4 kHz. TM locations that gave the best signal to noise ratios were the umbo, the anterior superior quadrant, the anterior inferior quadrant, and the posterior inferior quadrant (Figures 3, 4).

Figures 3 and 4 report TM displacement or velocity (respectively) versus sound pressure data for all six TM locations. Thick lines represent the frequencies for which the SNR was met. The posterior superior quadrant (Figure 3A, 4A) demonstrated the fewest frequencies for which the SNR was met, with only a few scattered frequencies that were acceptable. The anterior superior quadrant (Figure 3B, 4B) demonstrated acceptable SNR between 2500 and 8000 Hz. The lateral process of the malleus location (Figure 3C, 4C) had an acceptable SNR from 3000-4000 Hz. The umbo data (Figure 3D, 4D) revealed acceptable SNR from 2500-7000 Hz. The posterior inferior quadrant location (Figure 3E, 4E) had the most points which met the SNR criteria, which was 2000-8000 Hz. Finally, the SNR for the anterior inferior quadrant (Figure 3F, 4F) were found to be acceptable from 2500 to 8000 Hz.

**Figure 5:**
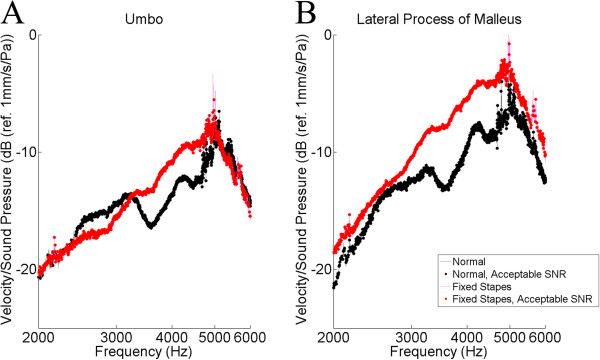
**Statistical analysis (*t*-test) of stapes footplate fixation versus normals.** The logarithm of tympanic membrane velocity measured at the umbo (**A**.) and the lateral process of the malleus (**B**.), normalized to background sound pressure and plotted against the frequency of sound stimulus. Following Bonferonni correction, no values reached statistical significance. SNR = Signal-to-noise ratio (see text).

**Figure 6:**
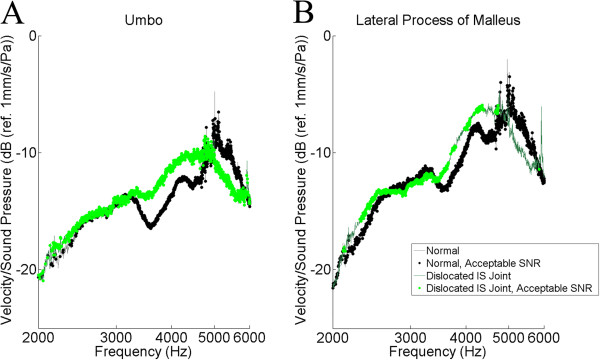
**Statistical analysis (*t*-test) of incudo-stapedial (IS) joint dislocation versus normals.** The logarithm of tympanic membrane velocity measured at the umbo (**A**.) and the lateral process of the malleus (**B**.), normalized to background sound pressure and plotted against the frequency of sound stimulus. Following Bonferonni correction, no values reached statistical significance. SNR = Signal-to-noise ratio (see text).

**Figure 7:**
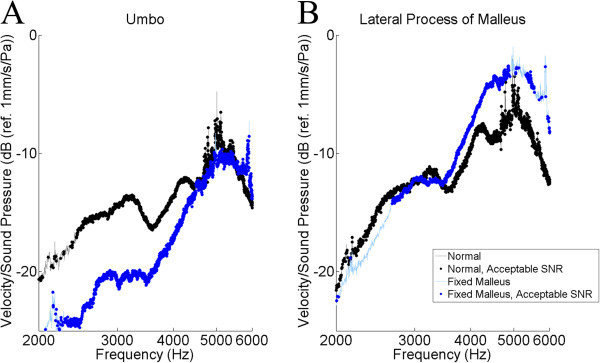
**Statistical analysis (*t*-test) of malleus head fixation versus normals.** The logarithm of tympanic membrane velocity measured at the umbo (**A**.) and the lateral process of the malleus (**B**.), normalized to background sound pressure and plotted against the frequency of sound stimulus. Following Bonferonni correction, no values reached statistical significance. SNR = Signal-to-noise ratio (see text).

The tracings for the umbo and the lateral process of the malleus were selected for further analysis (Figures 5, 6, 7). The results of the *t*-test and Bonferonni correction analysis are presented against the normals at the umbo and the lateral process of the malleus for the stapes footplate fixation (Figure 5), the dislocated IS joint (Figure 6), and for the malleus head fixation (Figure 7). Following Bonferonni correction, the results were not statistically significant, but trends were obtained. For both TM locations (Figure 5), the TM velocity for the stapes footplate fixation was higher than normals at most frequencies. For samples with a dislocated IS joint (Figure 6), TM velocity is comparable to normals between 2000 and 3000 Hz, is higher than normals between 3000 and 5000 Hz, then lower than normals between 5000 and 6000 Hz. The malleus head fixation group (Figure 7) was the only simulated ossicular pathology to demonstrate different patterns at the umbo and lateral process of the malleus measurement locations. At the umbo, the TM velocity is lower than normals up to 4500 Hz, then comparable to controls between 4500 and 6000 Hz. In contrast, at the lateral process of the malleus, the TM velocity is comparable to normals up to 3500 Hz, then is higher than normals between 3500 and 6000 Hz.

## Discussion

In this study, we have demonstrated that TM vibrations differ in our simulated ossicular pathologies compared with TM vibrations with a normal ossicular chain. This is best highlighted when TM vibrations were measured from 2 to 6 kHz frequencies.

Different TM locations provide different data quality in terms of signal-to-noise ratios (SNR). The poorest data was found for the posterior-superior quadrant, and the best data was found at the posterior-inferior quadrant. This may represent distinct mechanical properties of the TM at different locations, but the details of this proposition are purely speculative at present. Similar to results of Arechvo et al. [10], our data demonstrate considerable variability in TM vibration at different TM locations above 1500 Hz (compare panels A, B, C, D, E, and F of Figures 3 and 4). Studies such as ours emphasize the complexity of TM vibration and the role of basic science studies in further understanding the physical properties of the TM.

Our goal in this study was to assess the ossicular chain via TM vibrations. As such, we selected the TM locations closest to the ossicular chain (the umbo and lateral process of the malleus) for further (statistical) analysis. Since the TM is directly attached to the ossicular chain (malleus) at these locations, theoretically the TM movement would most closely reflect ossicular movement, and be less affected by pathologies affecting the TM itself (e.g. myringosclerosis). Tracings from Figures 3 and 4 demonstrate that both displacement and velocity offer similar separation of different ossicular pathologies, so we chose to use only one parameter (velocity) for our further analysis (Figures 5, 6, 7).

As expected based on the results with otosclerosis patients by Jakob et al. [5], the TM vibration was higher in the stapes fixation group compared with normals, above 2000 Hz (Figure 5). This lends support to the external validity of our model. Of note, measurements at the lateral process of the malleus produced better separation of the normal and stapes fixation curves. Since the umbo is further along the ossicular chain from the pathology (stapes footplate) compared to the lateral process of the malleus, perhaps manubrial bending [11] has contributed to the alteration in ossicular motion produced by the stapes footplate fixation. As such, the umbo vibration may actually be less sensitive to ossicular pathologies than the lateral process of the malleus. The lateral process of the malleus is easily visualized by the LDV, and may provide additional information to that provided by the umbo vibration.

IS dislocation produced a tracing not significantly different from the normals (Figure 6). Rosowski et al. [4] demonstrated higher magnitude of TM vibration in ears with interrupted ossicular chains below 1500 Hz. Unfortunately, our results did not demonstrate acceptable SNR below 1500 Hz. This may be due to the role of background noise, as our experimental setup did not use an isolation audiological booth. Due to the low velocities of TM motion at low frequencies, low frequency noise can impair measurements [12]. The use of an isolation sound booth may minimize this problem in the future.

Next, the malleus head fixation group demonstrated lower velocity than the normals when measured at the umbo, but higher velocity than the normals when measured at the lateral process of the malleus (Figure 7). This impaired mobility when measured at the umbo is consistent with similar trends found in a patient with a fixed malleus [3]. Interestingly, different patterns are detected at the umbo and the lateral process of the malleus. Again, this may be due to the role of manubrial bending, but limited conclusions can be drawn as the results did not reach statistical significance.

Although the tracings for the simulated ossicular pathologies were separated from the normal tracing, following Bonferonni correction the values did not reach statistical significance. This may be a reflection of our small sample size (n = 4). However, except for the IS dislocation group, the trends observed do confirm data achieved by previous investigators [3,5]. Larger sample sizes will be needed to better define and delineate differences between normals and ossicular pathologies, as there is probable individual variation in responses between patients which would become less relevant in a larger sample.

Obtaining reliable LDV measurements is technically challenging. The TM and EAC are relatively shielded from light by the pinna and EAC. To permit adequate light to be reflected back from the TM such that the video camera could capture the light and accurate localization of the laser on the TM could be obtained, we needed to remove the pinna and part of the lateral cartilaginous EAC. Previous literature [9] has described the use of reflective beads to optimize the amount of light reflected. We chose not to use reflective beads in order to create a TM model that could be transitioned into a future in vivo protocol, as reflective beads would not be appropriate with live human participants. The Polytec vibrometer detected that there was sufficient signal without reflective beads in order to make measurements; however, there likely would have been a better SNR if reflective beads were used. Next, the anterior EAC canal wall can be prominent, and thus impairs visualization and laser Doppler measurements from the anterior TM. For these temporal bones, an anterior canal wall canaloplasty was required to provide adequate visualization. Following our modifications, we were able to obtain reproducible tracings from all TM locations. This finding, in addition to the comparable nature of our results to those of previous investigators, serves as both internal and external validation of our methodology, and establishes the basis for future basic science and clinical LDV studies at our centre. We should highlight, however, potential future difficulties in our transition to live human subjects. Clearly, the modifications which were made to the cadavers would not be performed in a human study. This will necessitate further optimization of lighting and laser positioning to achieve reproducible results. Given our use of the anterior canaloplasty in this study, perhaps anterior TM measurements may not be possible in all human subjects. Note should be made that although a clinical hearing laser vibrometer is commercially available (Polytec GmbH, Waldbronn, Germany) that can be used in vivo, it does not provide unobstructed access to the entire TM via the EAC. Overcoming these technical challenges will be paramount in future experiments in our centre.

Previous literature [13] demonstrates that the cadaveric temporal bone is a valid model of middle and inner ear mechanics as measured by LDV. As LDV is a non-contact technique, the mechanical properties of the TM were not altered by our measurements. Therefore, the measured cadaveric TM vibrations in our study mimic the vibrations which would occur in a normal live human ear exposed to the same acoustic stimulus. Our cadaveric measurements in this study will permit an educated evaluation of live human TM vibrations in future studies.

This study also describes a useful model for the investigation of ossicular abnormalities in cadaveric temporal bones, a variant of the models described by Stasche et al. [14]. Fixation of the stapes footplate and the malleus head can be performed with a mastoidectomy, facial recess, and removal of the mastoid segment of the facial nerve. Adequacy of fixation is readily confirmed by ossicular palpation. Other ossicular manipulations, such as the IS joint dislocation in the present study, can also readily be performed and confirmed by inspection and palpation. This model is inexpensive, easy to perform for otolaryngologists, and uses commonly available materials (ethyl cyanoacrylate glue).

The motivation for this study was to evaluate the possibility of diagnosis of ossicular pathology in patients with a conductive hearing loss in the presence of an intact TM. Often patients undergoing an exploratory tympanotomy do not get a preoperative CT scan unless there is concern for cholesteatoma or if the patient has had prior ear surgery, as a CT scan would expose these patients to unnecessary radiation. Furthermore, ossicular abnormalities often cannot be diagnosed by CT. If ossicular pathologies could be diagnosed preoperatively with LDV, this would theoretically permit better surgical planning, and would limit the role of diagnostic exploratory tympanotomy. From the current study, LDV can suggest a diagnosis of ossicular abnormalities, but at present exploratory tympanotomy remains the gold standard for diagnosis.

## Conclusions

Laser Doppler Vibrometry measurements are challenging. However, once proper TM positioning and lighting have been obtained, LDV delivers consistent and reproducible results which characterize TM vibration responses to a sound stimulus. This modality holds great promise as a diagnostic tool to characterize the status of the ossicles in patients with a conductive hearing loss and an intact TM.

## 

**This manuscript was presented at:** Canadian Society of Otolaryngology - Head and Neck Surgery 66^th^ Annual Meeting, Fairmont Royal York Hotel, Toronto, Ontario, Canada. May 21, 2012.

## Competing interest

The authors have no actual or potential conflict of interest to disclose.

## Authors’ contributions

JB, HL, and SA designed the experiments. JB and SR collected and analyzed the data. JB, SR, HL, and SA wrote the manuscript. All authors discussed the results and implications and commented on the manuscript at all stages of preparation. All authors read and approved the final manuscript.
